# Impact of the Severities of Glaucoma on the Incidence of Subsequent Dementia: A Population-Based Cohort Study

**DOI:** 10.3390/ijerph17072426

**Published:** 2020-04-02

**Authors:** Fu-Hsuan Kuo, Jui-Fu Chung, Min-Yen Hsu, Chia-Yi Lee, Jing-Yang Huang, Ming-Ju Hsieh, Shun-Fa Yang

**Affiliations:** 1 Institute of Medicine, Chung Shan Medical University, Taichung 402, Taiwan; kfs0611@gmail.com (F.-H.K.); wchinyang@gmail.com (J.-Y.H.); 2 Neurological Institute, Taichung Veteran General Hospital, Taichung 407, Taiwan; 3 Radiology Division, Chiayi Branch, Taichung Veteran General Hospital, Chiayi 600, Taiwan; jeff110175@gmail.com; 4 Department of Ophthalmology, Chung Shan Medical University Hospital, Taichung 402, Taiwan; my.scott.hsu@gmail.com; 5 School of Medicine, Chung Shan Medical University, Taichung 402, Taiwan; 6 Department of Ophthalmology, Show Chwan Memorial Hospital, Changhua 500, Taiwan; ao6u.3msn@hotmail.com; 7 Cancer Research Center, Changhua Christian Hospital, Changhua 500, Taiwan; 8 Graduate Institute of Biomedical Sciences, China Medical University, Taichung 404, Taiwan

**Keywords:** glaucoma, dementia, epidemiology, severity, Alzheimer disease, Parkinson disease

## Abstract

The aim of the present study was to survey the relationship between the severity of glaucoma and subsequent dementia using the National Health Insurance Research Database (NHIRD) in Taiwan. Subjects with glaucoma were selected into the study group after an exclusion process, and each subject in the study group was propensity score-matched to another non-glaucoma patient that constituted the control group. The Cox proportional hazard regression that considered multiple potential risk factors of dementia was used to yield the adjusted hazard ratios (aHR) of dementia in different severities of glaucoma. There were 1185 (5.63 percent) subjects in the study group and 1119 (5.32 percent) patients in the control group that developed dementia. After adjusting for multiple confounders, there were no differences in the rate of any dementia (aHR: 0.961, 95% CI: 0.886–1.043, *p* = 0.3443), vascular dementia (aHR: 0.928, 95% CI: 0.846–1.018, *p* = 0.1154), Alzheimer’s disease (aHR: 1.018, 95% CI: 0.761–1.362, *p* = 0.9025) or Parkinson’s disease (aHR: 1.021, 95% CI: 0.886–1.176, *p* = 0.7744) between the study and the control groups. Regarding the disease severity of glaucoma, no difference was found in any type of dementia whether the glaucoma patients received less than two medical treatments, received more than two medical treatments, received drainage surgeries or received destructive surgeries. In conclusion, the different severities of glaucoma do not alter the incidence of subsequent dementia.

## 1. Introduction

Dementia refers to the progressive loss of cognitive function that can retard occupational, social, or domestic functioning [1]. To date, more than five percent of the population suffer from dementia, in which the prevalence rises to nearly 10 percent in developed countries [2,3]. The causes of dementia include advanced age, the presence of vascular disease and some genetic variation [1]. In addition to the loss of social and cognitive functions, dementia also leads to a higher mortality rate in certain situations compared to those without such neurodegenerative disorders [4,5].

The associated co-morbidities and environmental risk factors are not uncommon in subjects with dementia [1,6]. Systemic vascular diseases are significantly correlated to dementia development [7], and cerebrovascular disease itself can lead to the onset of vascular dementia (VD) [1,8]. The presence of diabetes mellitus (DM) was associated with vascular dementia and Alzheimer’s disease (AD) in a previous study [9]. while advanced age and gait disturbance are predictors to Parkinson’s disease (PD) with cognitive defects [10]. Regarding the sensory organs, the presence of dementia like AD and PD relates to the occurrence of sensorineural hearing loss (SNHL) [11,12]. Moreover, persistent visual loss, including those diagnosed with age-related macular degeneration (AMD), has been proven to result in the development of subsequent dementia [13,14].

Glaucoma is a neurodegenerative eye disease featuring optic neuropathy and stands as the most common cause of legal blindness in developed countries [15,16]. A previous study demonstrated that glaucoma and Alzheimer’s disease could co-exist in the elderly [17], which may be due to the similar neurodegenerative features [18]. On the other hand, the degree of glaucoma is various; some can be easily controlled by single medications, while destructive surgery may be warranted for refractory cases [15,19]. Since the glaucoma severity indicates different extents of optic nerve damage and vulnerability, the degree of glaucoma severity might also relate to the subsequent dementia onset, which has seldom been elucidated before.

The purpose of the current study is to survey whether the severity of glaucoma, based on the treatment patients received in a range of time, altered the incidence of subsequent dementia, using the National Health Insurance Research Database (NHIRD) in Taiwan. Moreover, the effect of different glaucoma subtypes and other potential risk factors of dementia were included in the multivariable analysis.

## 2. Materials and Methods

### 2.1. Ethics Declaration and Data Source

The current study adhered to the declaration of Helsinki in the 1964 and its late amendment and was also approved by the Institutional Review Board of Chung Shan Medical University Hospital (Project identification code: CS-17075) and the National Health Insurance Administration of Taiwan. The data source of the current study was from the Longitudinal Health Insurance Database 2005 version (LHID), which enrolled about two million subjects derived from the NHIRD. Since the data in LHID 2005 version were randomly sampled at the year 2005, near the mid-point of the whole follow-up interval (from 1997 to 2016), this version presented the whole study population better than other versions. Since the population in LHID was randomly extracted from the NHIRD via the computer program provided by the National Health Insurance Administration of Taiwan, there was rarely selection bias in the LHID. The information available from the LHID included the demographic data, the socioeconomic condition, the residence of subjects, the International Classification of Diseases-Ninth Revision (ICD-9), the International Classification of Diseases-Tenth Revision (ICD-10) and the medications used for the study subjects. The interval of LHID ranged from January 1 1997 to December 31 2016, spanning a total interval of 16 years.

### 2.2. Subject Selection

The patients in LHID were regarded as developing glaucoma (i.e., the inclusion criteria) if their medical data indicated: (1) the receipt of a diagnosis of glaucoma according to the ICD-9/ICD-10 codes; (2) the diagnosis of glaucoma was made by an ophthalmologist; (3) the receipt of an optical coherence tomography exam or visual field test before the glaucoma diagnosis; and (4) an age from 20 to 100 years. The index date was set as two years after the date of glaucoma diagnosis to strength the correlation between exposure and outcome. On the other hand, the subsequent exclusion criteria were employed for the whole study population to erase certain factors that would influence the analysis prominently: (1) the diagnosis of legal blindness before the index date; (2) the diagnosis of ophthalmic malignancy before the index date; (3) the receipt of eyeball removal surgery before the index date; and (4) the diagnosis of severe ocular trauma before the index date. Then, the patients in the study group would be further excluded if any of the following factors existed: (1) index date before 2005; (2) age less than 20 or more than 100 years; (3) death before the index date; and (4) the occurrences of outcome (demonstrated in the following section) before the index date. After that, each patient in the study group was propensity score-matched with another non-glaucoma patient considering the demographic data, socioeconomical status and co-morbidities, the latter of which were collectively defined as the control group. For the subgroup analysis, subjects in the study group were divided into those who diagnosed as open angle glaucoma (OAG), normal tension glaucoma (NTG) and angle closure glaucoma (ACG). The study group was further categorized into; (1) those that received less than two types of medical treatment within two years after the date of glaucoma diagnosis; (2) those that received more than two types of medical treatment within two years after the date of glaucoma diagnosis; (3) those that received the drainage surgery, including trabeculectomy and drainage device implant, within two years after the date of glaucoma diagnosis; and (4) those that received destructive surgery, including cyclocoagulation and cryotherapy, within two years after the date of glaucoma diagnosis. All these subgroups in the study group were compared to the control group regarding the development of dementia in the analysis model.

### 2.3. Main Outcome Measurement

The development of dementia was the primary outcome for the current study, which including the following conditions: (1) the diagnosis of VD according to ICD-9/ICD-10 codes; (2) the diagnosis of AD according to ICD-9/ICD-10 codes; and (3) the diagnosis of PD with concurrent dementia according to ICD-9/ICD-10 codes. These primary outcomes were analyzed separately and summed up to calculate the total amount of dementia events during the whole study period.

### 2.4. Demographic Variables and Co-Morbidities

To make the status of each participant in the current study more similar, the subsequent factors were considered in both the matching process and the multivariable regression analysis: age, gender, education level, marital status, hypertension, DM, ischemic heart diseases, hyperlipidemia, congestive heart failure, peripheral vascular disease, cerebrovascular disease, SNHL, AMD and hemiplegia or paraplegia. We longitudinally followed the patient’s condition from the index date until the date of any type of dementia diagnosis, or until the last date of data collection from the LHID/NHIRD, which meant December 31, 2016.

### 2.5. Statistical Analysis

The software SAS version 9.4 (SAS Institute Inc, NC, USA) was applied for all the statistical analyses used in the current study. Firstly, the propensity score-matching process with a 1:1 ratio (nearest greedy, caliper = 0.01). The absolute standardized difference (ASD) in the propensity score-matching process was also calculated and a value less than 0.1 indicated no difference between the study and control groups. After that, both the incidence relative risk (IRR) and the 95% confidence intervals (CI) were calculated via the Poisson regression model. Then the Cox proportional hazards regression was applied to compute the adjusted hazard ratios (aHR) of dementia for glaucoma and other potential risk factors via incorporating age, gender, education level, marital status and related comorbidities into the multivariable analysis. The Kaplan–Meier curves were plotted to illustrate the cumulative probability of each type of dementia between the study and control groups, while the log-rank test was used to investigate whether a significant difference existed between the two different survival curves. After that, we analyzed the aHR of each type of dementia including VD, AD and PD in glaucoma with different severities (according to the treatment they received) compared to corresponding non-glaucoma participants. Further to this, we compared the aHR of dementia in different glaucoma subtypes. Because more than 98 percent of subjects in Taiwan are Han Taiwanese, the ethnicity was not considered a covariate in the current study using the LHID of Taiwan. Statistical significance was set at *p* < 0.05, while a *p* value of less than 0.0001 was shown as *p* < 0.0001.

## 3. Results

**Figure 1:**
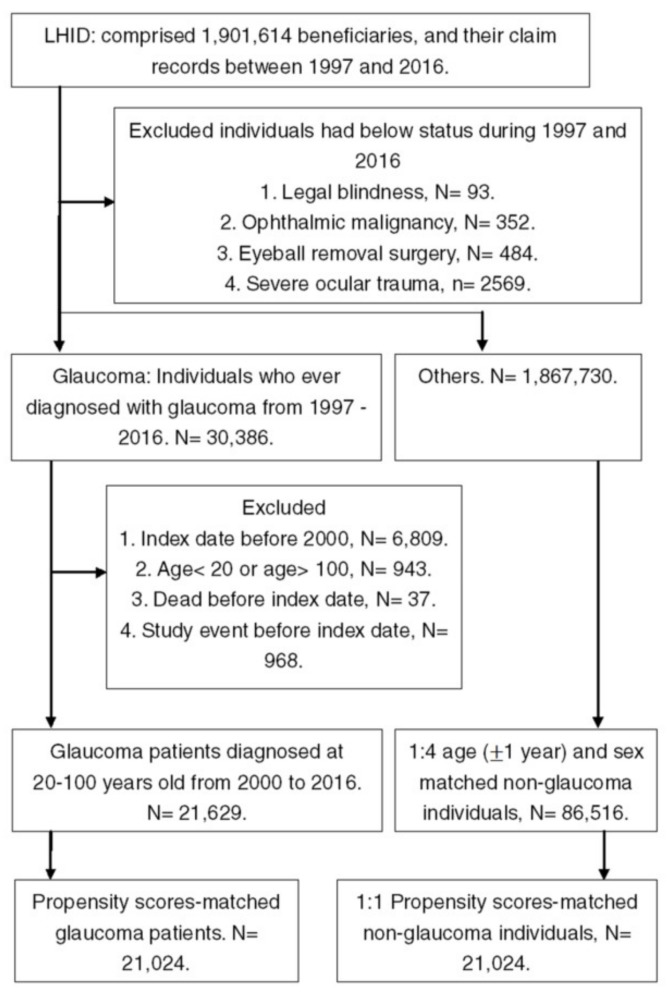
The flowchart of subject selection. LHID: Longitudinal Health Insurance Database 2005 version; N: number.

A total of 21,024 patients with glaucoma were enrolled in the study group, while the same numbers of subjects were included in the control group. The process of subject selection is shown in Figure 1. There were no differences concerning the distribution of demographic data, socioeconomic status and co-morbidities between the study and control groups according to the ASD values (Table 1).

**Figure 2:**
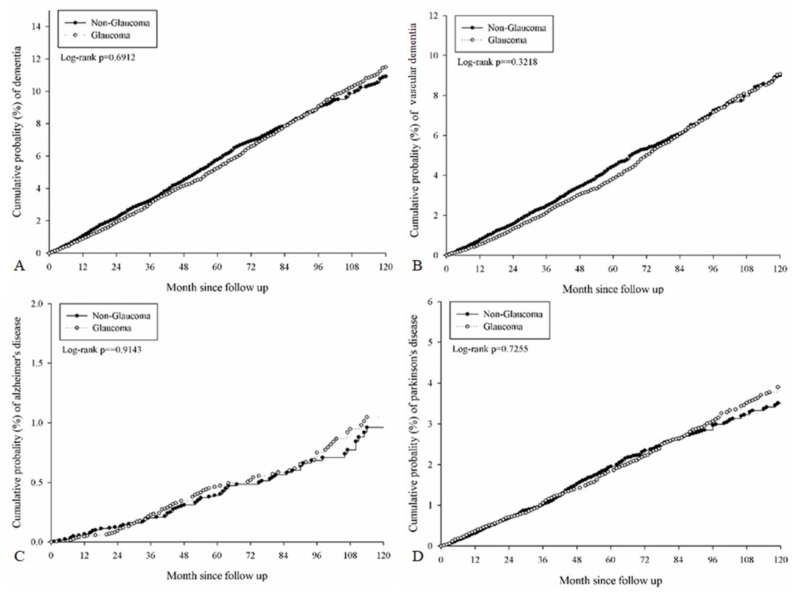
The Kaplan–Meier curves for the cumulative probability of each dementia. (**A**) Cumulative probability of any dementia. (**B**) Cumulative probability of vascular dementia. (**C**) Cumulative probability of Alzheimer’s disease. (**D**) Cumulative probability of Parkinson’s disease.

After a follow-up period of up to 16 years, 1185 (5.63 percent) subjects in the study group and 1119 (5.32 percent) patients in the control group developed any type of dementia (Table 2). There were no significant differences in the incidence rate of any type of dementia (crude IRR: 0.99, 95% CI: 0.91–1.07), VD (crude IRR: 0.96, 95% CI: 0.88–1.05), AD (crude IRR: 1.05, 95% CI: 0.79–1.41) or PD (crude IRR: 1.03, 95% CI: 0.89–1.18) between the study and the control groups (Table 2). After adjusting for multiple risk factors, there were still no differences in the incidence rate of any type of dementia (aHR: 0.961, 95% CI: 0.886–1.043, *p* = 0.3443), VD (aHR: 0.928, 95% CI: 0.846–1.018, *p* = 0.1154), AD (aHR: 1.018, 95% CI: 0.761–1.362, *p* = 0.9025) or PD (aHR: 1.021, 95% CI: 0.886–1.176, *p* = 0.7744) between the study and the control groups (Table 3), and the cumulative probabilities of each dementia were all insignificant (Log-rank *p* > 0.05, Figure 2). The other risk factors that were associated with the development of dementia included: age older than 50–60 years (all *p* < 0.05), hypertension (*p* = 0.0013), DM (*p* < 0.0001), ischemic heart disease (*p* = 0.0013), congestive heart failure (*p* = 0.0077), cerebrovascular disease (*p* < 0.0001), SNHL (*p* = 0.0155) and hemiplegia or paraplegia (*p* = 0.0003) (Table 3).

In the subgroup analysis of the disease severity of glaucoma according to the treatment arranged, no difference was found for whether the glaucoma patients received less than two types of medical treatment, received more than two types of medical treatment, received drainage surgeries or received destructive surgeries in any type of dementia and the three subtypes of dementia (Table 4). Regarding the glaucoma subtype, the OAG, NTG and ACG did not elevate the risk of dementia development, except a mild protective effect of OAG on the development of VD (Table 5).

## 4. Discussion

In the current study, we found that the presence of glaucoma did not elevate the chance of dementia occurrence. Moreover, the different severity and progression of glaucoma lead to insignificant influence of all dementia events including VD, AD and PD. On the other hand, certain cardiovascular, metabolic and neurological diseases prominently elevate the risk of subsequent dementia development.

There are multiple etiologies that contribute to dementia that can be roughly divided in to “neurodegenerative”, “non-neurodegenerative” or mixed types [1,20], while the decline of cognitive function is the universal character of all dementia [6]. The advanced age accounts for the majority of involution dementia worldwide [1], while depression may play a role in general dementia development [21]. For specific pathophysiology of dementia, the direct damage to brain tissue via ischemic stroke or intracranial hemorrhage is the mechanism of VD [22], and evensmall vessel disease can cause following VD episodes [23]. The AD, featured with the accumulation of both the protein beta-amyloid and tau tangles, leads to synapse decline and neuron death progressively [5]. Meanwhile, PD is caused by the deficiency of levodopa and is commonly associated with speech and movement defects [24]. Concerning the vision and dementia, the patients with moderate to severe visual impairment are at higher risk of developing dementia compared to those with normal vision [13]. The retinal neural degeneration as well as impairment of microvasculature were linked to the dementia episode [25,26]. On the contrary, the pathway of neuron death is different between certain dementia types, like AD or VD, and some ocular diseases that would lead to permanent visual loss, including glaucoma and AMD [1,15,27]. Although the same risk factors of advanced age and vascular defects were illustrated [5,15,22], glaucoma possesses other predisposing factors like ocular inflammation or the closure of the gonio angle [28,29]. As the number of dementia events has progressively increased, this is a huge socioeconomical issue now and into the future [30]. It is important to find out whether ocular diseases with severe visual impairment lead to a higher rate of dementia occurrence.

Several researchers have discussed the relationship between dementia and eye diseases [17,31,32]. In one study, the percentage of glaucoma was significantly higher in those with dementia, and vice versa [17]. Another retrospective study illustrated that OAG was associated with AD but not PD [31]. In the current study, the severity of glaucoma did not enhance the risk of following dementia, which is a relatively novel finding to our knowledge since studies rarely evaluate the relationship between glaucoma severity and dementia development. Since both glaucoma and dementia are neurodegenerative diseases [1,15], it is reasonable that more severe glaucoma should correlate to a higher rate of dementia. However, the results of the current study argue against that theory, since the existence of glaucoma did not elevate the chance of dementia. There are two possible explanations for this phenomenon. Firstly, the age of glaucoma onset is relatively earlier than the age of dementia occurrence, for which the follow up period of the current study might be inadequate [1,15]. On the other hand, the current study included multiple covariates that would lead to the development of dementia in the regression analysis, while the previous study only compared the relationship between glaucoma and dementia with a small study population [17]. Further study is suggested to investigate this issue more completely.

In the subgroup analysis of the current study, no significant difference is found about the rate of dementia in different subtypes of glaucoma compared to propensity score-matching subjects without glaucoma. The mechanisms of the three subtypes of glaucoma included in the current study are different; the ACG is caused by the acute elevation of intraocular pressure, while the OAG and the NTG are resulted from long-term ocular hypertensive status and possible low-perfusion condition [28,33,34]. That the incidence of dementia did not elevate in all subtypes of glaucoma may further indicate the different pathophysiologies of neurological degradation between the brain and retina, which is supported by the findings of the current study.

Regarding other risk factors of dementia, several cardiovascular diseases lead to a higher rate of dementia development, which may due to the correlation of vessel damage and brain degeneration proven in previous research [1,7,8,23]. Besides, DM is associated with the occurrence of cardiovascular disease and can also lead to certain types of dementia, which correlated to the finding of the current study [9]. The SNHL is also a type of neurodegenerative disease and is proven to correlate to cognitive decline and dementia by previous studies [35]. Furthermore, older age is also a well-established risk factor for dementia in which the current study further confirmed this viewpoint [36].

Certain limitations still exist in the current study. The nature of claimed data research made it hard to access the exact severity of glaucoma and family history of dementia. In addition, one individual may possess multiple types of dementia (i.e., combined AD and VD) and we only selected the first event of dementia as the outcome achievement, thus those with multiple dementias cannot be found and evaluated. Moreover, the ICD9/ICD-10 codes “unspecific glaucoma” may include any type of glaucoma and account for a considerable number of glaucoma diagnoses in clinical practice, thus some patients cannot be categorized into the subgroup analysis evaluating the effect of OAG, NTG and ACG. Nevertheless, the gross analysis between the glaucoma population and the non-glaucoma population revealed insignificant differences, which is similar to the subgroup analysis. Consequently, the influence of this rough diagnosis process might not be prominent.

## 5. Conclusions

In conclusion, the different severities of glaucoma do not alter the incidence of subsequent dementia after adjusting multiple potential risk factors. Furthermore, the subtype of glaucoma and the glaucoma duration also lead to an insignificant effect on dementia development. Further large-scale prospective studies with adequate patient numbers to reveal whether multiple neurodegenerative ophthalmic diseases would change the probability of dementia occurrence is mandatory.

## Figures and Tables

**Table 1:** Characteristics among study cohorts in 1:1 propensity score-matching populations.

Characteristics	Study	Control	ASD
N	21024	21024	
Age			0.008
20–30	1109 (5.27%)	1073 (5.10%)	
30–40	1717 (8.17%)	1632 (7.76%)	
40–50	3112 (14.8%)	2976 (14.16%)	
50–60	4708 (22.39%)	4672 (22.22%)	
60–70	5126 (24.38%)	5183 (24.65%)	
70–80	3752 (17.85%)	3919 (18.64%)	
80–100	1500 (7.13%)	1569 (7.46%)	
Sex			0.055
Male	10,494 (49.91%)	10,543 (50.15%)	
Female	10,530 (50.09%)	10,481 (49.85%)	
Education			0.037
<6	7467 (35.52%)	7556 (35.94%)	
6–9	3104 (14.76%)	3047 (14.49%)	
9–12	7350 (34.96%)	7304 (34.74%)	
> = 12	3103 (14.76%)	3117 (14.83%)	
Marry			0.001
Not marriage	3464 (16.48%)	3322 (15.80%)	
Marriage	15,046 (71.57%)	15,197 (72.28%)	
Divorce	1123 (5.34%)	1087 (5.17%)	
Death of spouse	1391 (6.62%)	1418 (6.74%)	
Co-morbidities			
Hypertension	7692 (36.59%)	7918 (37.66%)	0.022
DM	4858 (23.11%)	4883 (23.23%)	0.003
Ischemic heart diseases	1818 (8.65%)	1863 (8.86%)	0.008
Hyperlipidemia	4574 (21.76%)	4603 (21.89%)	0.003
Congestive heart failure	607 (2.89%)	632 (3.01%)	0.007
Peripheral vascular disease	331 (1.57%)	342 (1.63%)	0.004
Cerebrovascular disease	1399 (6.65%)	1385 (6.59%)	0.003
SNHL	101 (0.48%)	97 (0.46%)	0.003
AMD	479 (2.28%)	471 (2.24%)	0.003
Hemiplegia or paraplegia	136 (0.65%)	107 (0.51%)	0.018

ASD: absolute standardized difference; N: number; DM: diabetes mellitus; SNHL: sensorineural hearing loss; AMD: age-related macular degeneration

**Table 2:** Dementia events in the propensity score-matching study populations.

Study Event	Study		Control		Crude IRR (95% CI)
	Event Number/ Person-months	Incidence Rate, per 1000 PMs (95% CI)	Event Number/ Person-Months	Incidence Rate, per 1000 PMs (95% CI)	
Any dementia	1185/1222688	9.69 (9.16–10.26)	1119/1138846	9.83 (9.27–10.42)	0.99 (0.91–1.07)
VD	906/1234948	7.34 (6.87–7.83)	878/1149290	7.64 (7.15–8.16)	0.96 (0.88–1.05)
AD	97/1262946	0.77 (0.63–0.94)	86/1177323	0.73 (0.59–0.90)	1.05 (0.79–1.41)
PD	405/1249010	3.24 (2.94–3.57)	368/1166108	3.16 (2.85–3.50)	1.03 (0.89–1.18)

IRR: incidence relative risk; PMs: person-months; CI: confidence intervals; VD: vascular dementia; AD: Alzheimer’s disease; PD: Parkinson’s disease

**Table 3:** Cox regression for estimates of the adjusted hazard ratios of dementia.

Parameters	aHR (95% CI)	*p* Value
Glaucoma for all dementia (ref = Control)	0.961 (0.886–1.043)	0.3443
Glaucoma for VD	0.928 (0.846–1.018)	0.1154
Glaucoma for AD	1.018 (0.761–1.362)	0.9025
Glaucoma for PD	1.021 (0.886–1.176)	0.7744
Age (ref = 50–60)		
20–30	0.106 (0.033–0.341)	0.0002
30–40	0.099 (0.036–0.269)	<0.0001
40–50	0.293 (0.185–0.462)	<0.0001
60–70	4.681 (3.832–5.717)	<0.0001
70–80	11.244 (9.239–13.683)	<0.0001
80–100	18.864 (15.331–23.210)	<0.0001
Sex (ref= Male)		
Female	1.064 (0.971–1.165)	0.1826
Education (ref = 9–12 years)		
<6	0.996 (0.894–1.110)	0.9492
6–9	0.870 (0.749–1.009)	0.0663
> = 12	0.906 (0.762–1.076)	0.2612
Marry (ref= not marriage)		
Marriage	0.975 (0.775–1.226)	0.8276
Divorce	1.112 (0.823–1.503)	0.4899
Death of spouse	1.097 (0.856–1.406)	0.4642
Co-morbidities		
Hypertension	1.160 (1.060–1.269)	0.0013
DM	1.232 (1.123–1.351)	<0.0001
Ischemic heart diseases	1.196 (1.073–1.335)	0.0013
Hyperlipidemia	1.003 (0.911–1.106)	0.9457
Congestive heart failure	1.258 (1.063–1.490)	0.0077
Peripheral vascular disease	1.204 (0.952–1.522)	0.1204
Cerebrovascular disease	1.580 (1.410–1.771)	<0.0001
SNHL	1.543 (1.086–2.192)	0.0155
AMD	0.981 (0.794–1.213)	0.8596
Hemiplegia or paraplegia	1.849 (1.323–2.585)	0.0003

aHR: adjusted hazard ratios; CI: confidence intervals; VD: vascular dementia; AD: Alzheimer’s disease; PD: Parkinson’s disease; DM: diabetes mellitus; SNHL: sensorineural hearing loss; AMD: age-related macular degeneration;

**Table 4:** Correlation between glaucoma severities and the development of dementia events.

Events	aHR (95% CI) for Dementia				
	Control *N* = 21024	No more than Two Medications *N* = 16690	More than Two Medications *N* = 3665	Drainage Surgery *N* = 582	Destructive Surgery *N* = 87
Any dementia	Reference	0.930 (0.851–1.017)	1.043 (0.913–1.192)	1.186 (0.886–1.589)	0.820 (0.389–1.727)
VD	Reference	0.886 (0.800–0.981)	1.049 (0.903–1.218)	1.168 (0.840–1.623)	0.736 (0.305–1.776)
AD	Reference	1.027 (0.751–1.404)	1.044 (0.647–1.683)	0.575 (0.141–2.338)	1.677 (0.232–12.125)
PD	Reference	0.989 (0.849–1.153)	1.131 (0.902–1.419)	1.183 (0.706–1.984)	0.366 (0.051–2.606)

aHR: adjusted hazard ratios; CI: confidence intervals; N: number; VD: vascular dementia; AD: Alzheimer’s disease; PD: Parkinson’s disease

**Table 5:** Correlation between type of glaucoma and the development of dementia events.

Events	aHR (95% CI) for Dementia			
	Control	OAG	NTG	ACG
Any dementia	Reference	0.874 (0.761–1.004)	1.169 (0.977–1.399)	0.913 (0.814–1.024)
VD	Reference	0.833 (0.709–0.977)	1.073 (0.869–1.325)	0.899 (0.791–1.023)
AD	Reference	0.825 (0.490–1.390)	0.928 (0.449–1.916)	0.927 (0.622–1.381)
PD	Reference	0.982 (0.780–1.236)	1.440 (1.086–1.909)	0.962 (0.786–1.176)

aHR: adjusted hazard ratios; CI: confidence intervals; OAG: open angle glaucoma; NTG: normal tension glaucoma; ACG: angle closure glaucoma; VD: vascular dementia; AD: Alzheimer’s disease; PD: Parkinson’s disease
